# Unusual course of generalized lymph node primary plasmacytoma in a patient with Sjögren’s syndrome: a case report

**DOI:** 10.1186/s13256-017-1266-7

**Published:** 2017-04-20

**Authors:** Vadim R. Gorodetskiy, Natalya A. Probatova, Vladimir I. Vasilyev

**Affiliations:** 1 Department of Intensive Methods of Therapy, V.A. Nasonova Research Institute of Rheumatology, Russian Academy of Medical Sciences, Kashirskoye shosse 34A, Moscow, 115522 Russia; 2 Department of Pathology, N.N. Blokhin Russian Cancer Research Center, Russian Academy of Medical Sciences, Kashirskoye shosse 24, Moscow, 115478 Russia

**Keywords:** Plasmacytoma, Lymph node, Sjögren’s syndrome, Spontaneous regression

## Abstract

**Background:**

Primary lymph node plasmacytoma is a rare disease that typically involves lymph nodes of the neck. In only 15% of cases is the disease generalized. Here, we present a case of generalized lymph node plasmacytoma in a patient with Sjögren’s syndrome with an unusual course.

**Case presentation:**

A 48-year-old white woman presented to our hospital with enlargement of groups of lymph nodes, liver, and spleen. Her medical history was consistent with a 12-year course of Sjögren’s syndrome. Blood and urine immunochemistry showed a massive (72 g/l) M-gradient formed from immunoglobulin Aκ in the serum and monoclonal free κ-type light chains in her urine. A skeletal X-ray revealed no bone destruction. Cytological and histological bone marrow assays showed no signs of plasma cell infiltration. The microarchitecture of her neck and inguinal lymph nodes was destroyed. Only small remnants of B cell follicles were found, while the interfollicular areas were expanded and infiltrated by CD138, MuM1, CD43, and﻿ IgAκ-positive plasma cells. After nine cycles of doxorubicin, cyclophosphamide, vincristine, and prednisolone chemotherapy, complete remission was achieved. However, the lymphoma relapsed 3 months later, with histological verification in her femoral lymph node. Despite the absence of subsequent adequate therapy, she gradually achieved complete remission of plasmacytoma with the disappearance of paraproteins.

**Conclusions:**

Currently, primary lymph node plasmacytoma is generally considered a nodal marginal zone lymphoma with an extensive plasmacytic differentiation. In our case, despite the critical histological and immunohistochemical evaluation of three lymph node biopsies from different anatomical areas at different times, no signs of nodal marginal zone lymphoma were found. An 18-year follow-up of our patient with primary lymph node plasmacytoma demonstrated an extremely unusual clinical course. Initially, primary lymph node plasmacytoma was refractory to chemotherapy. However, subsequently, she underwent a complete spontaneous remission of plasmacytoma.

## Background

Primary lymph node plasmacytoma (PLNP) or plasmacytic lymphoma (in the Kiel classification [1]) is a rare lymphoid tumor consisting of neoplastic plasma cells typically involving lymph nodes. PLNP has an indolent clinical course without progression to plasma cell myeloma [2–4]. In most cases, PLNP is localized in the cervical lymph nodes. Generalized lymph node involvement is observed rarely. Approximately 80 cases of PLNP are described in the literature, either as sporadic cases or as a small series [2–20].

Spontaneous regression (SR) of cancer is the complete or partial disappearance of a malignant tumor without treatment or in the presence of therapy that is considered inadequate to exert a significant influence on neoplastic disease [21]. This phenomenon has been reported in different indolent non-Hodgkin's lymphomas with a frequency of 10 to 20% [22]. In the literature, we found only one case of SR of an extramedullary plasmacytoma (EMP) in the soft tissues [5]. As far as we know, the spontaneous remission of PLNP is not described.

Here, we present a case of generalized PLNP with an unusual course in a patient with Sjögren’s syndrome (SS). PLNP had been refractory to chemotherapy initially, but later underwent a complete spontaneous remission.

## Case presentation

A 48-year-old white woman presented to our hospital with enlargement of all groups of peripheral lymph nodes. Lymph nodes formed conglomerates in her left supraclavicular area (up to 6.0 cm in size) with trachea compression and stridor development, and conglomerates in her left femoral-inguinal area (up to 5.0 cm in size) with development of lymphostasis in her left leg. No systemic signs (fever, night sweats, and weight loss) developed. Her medical history was consistent with a 12-year course of SS. At the age of 36, she presented with recurrent polyarthralgia. Serological testing revealed rheumatoid factor at a 1:512 titer (Waaler-Rose test). Subsequently, she developed recurrent parotitis, dry eyes, dry mouth, and recurrent purpura. A contrast X-ray study of her parotid gland indicated parenchymal parotitis. A labial minor salivary gland biopsy showed marked focal lymphocytic sialadenitis with a focus score of 4 (>50 lymphocytes in the 4 mm^2^ tissue sample). Schirmer's test was positive (<1 mm in 5 minutes). Her anti-Ro/SS-A antibody level was 130 U/ml (normal range <5.0 U/ml). Based on the clinical, serological, and pathological features of SS and the absence of radiographic changes of rheumatoid arthritis, primary SS was diagnosed. Three years before admission, an increase was noted in her left supraclavicular lymph nodes. However, she refused to undergo an examination.

At the time of admission, a complete blood count showed a reduction of her hemoglobin level to 89 g/l. The remaining hemogram parameters were within normal limits. Blood chemistry revealed an increase of total protein to 120 g/l. Her serum lactate dehydrogenase level was normal, and her blood viscosity was 7.9 mPa second (normal range <5.3 mPa second). Antibodies to human immunodeficiency virus (HIV) were absent.

Blood immunochemistry studies revealed a massive M-gradient formed from IgAκ. Her serum monoclonal IgAκ paraprotein level was 72 g/l. Her polyclonal immunoglobulin G (IgG) and immunoglobulin M (IgM) levels were reduced. A urine electrophoresis detected trace proteinuria with serum Aκ paraproteins and monoclonal free κ-type light chains, indicating Bence-Jones (BJ) proteins.

Chest and abdominal computed tomography revealed multiple enlarged lymph nodes of her upper mediastinum, lung roots, abdominal cavity, and retroperitoneal space (merging into conglomerates of up to 6.5 cm in size), as well as enlargement of her liver and spleen. A skeletal X-ray revealed no bone destruction. Her myelogram revealed 1.6% plasma cells and 7.6% lymphocytes. Bone marrow histology showed no signs of plasma cell or lymphocyte infiltration.

**Fig. 1:**
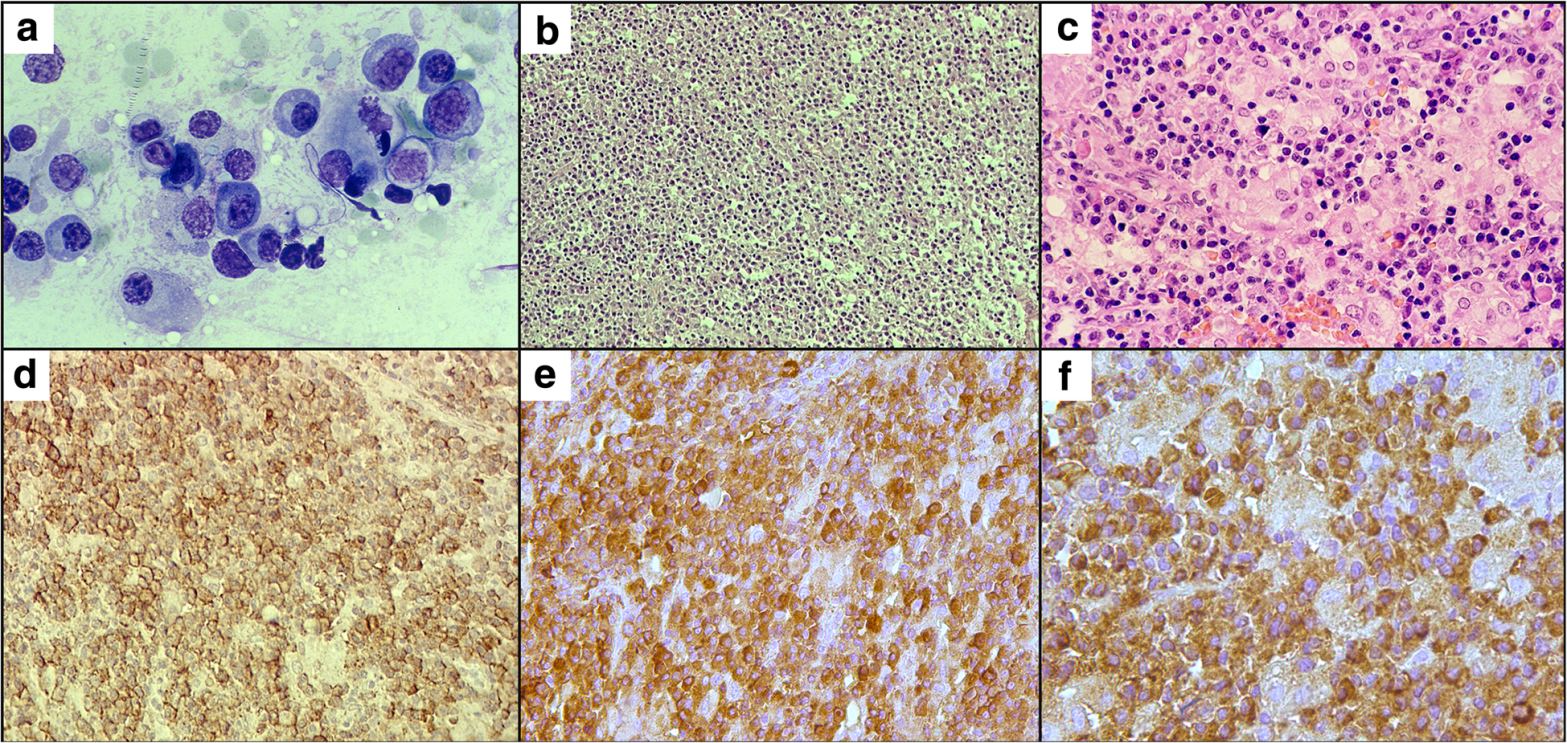
**a** The imprint cytology of a lymph node. Tumor infiltration of plasma cells is noted, along with several cells with distinct signs of atypia. Romanowsky-Giemsa staining, ×630. **b** Diffuse proliferation of plasma cells in lymph nodes. Hematoxylin and eosin stain, ×200. **c** Plasma cells with single lymphocytes, macrophages, and epithelioid histiocytes forming granuloma-like structures. Hematoxylin and eosin stain, ×400. **d** Plasma cells expressing CD138, ×400. **e** Monomorphic expression of IgA by plasma cells, ×400. **f** Monomorphic expression of kappa light chain by plasma cells, ×630

A diagnostic biopsy of her cervical and inguinal lymph nodes was performed. The imprint cytology of lymph nodes showed plasma cells, some with distinct atypia (Fig. 1a). The same findings were revealed in the histological results of her neck and inguinal lymph nodes. The microarchitecture of the nodes was destroyed. Only small remnants of B cell follicles were found, while the interfollicular areas were expanded and infiltrated by plasmacytically differentiated cells (Fig. 1b). These cells were positive for CD138 (Fig. 1d), MuM1, and CD43 and expressed IgAκ (Fig. 1e, f). Several plasma cells were positive for CD79a, and some of the plasma cells co-expressed CD20. The plasmacytic infiltration remained negative for CD56, VS38c, BCL6, BCL2, cyclin-D1, and CD23. Germinal centers (CD10+) were not detectable. The proliferative rate (Ki67) reached almost 10%. Plasma cells were negative for human herpesvirus type 8 (HHV-8) and for Epstein-Barr virus (EBV)-encoded small RNA (in situ hybridization). Among the plasma cells, single macrophages and epithelioid histiocytes were noted, represented by granuloma-like small structures (Fig. 1c).

Based on the examination data, generalized lymph node plasmacytoma was diagnosed. Our patient received nine cycles of doxorubicin, cyclophosphamide, vincristine, and prednisolone (CHOP) chemotherapy. Restaging showed complete remission. However, immunochemical serum and urine studies revealed a residual M-gradient, which was identified by immunofixation as Aκ, indicating residual disease. Three months after completion of chemotherapy the lymphoma relapsed. A 38.0 °C fever developed with rapid enlargement of all groups of lymph nodes (and the formation of conglomerates of up to 6.0 cm in size) and an increase of IgAκ paraprotein to 37.5 g/l. The paraprotein showed rheumatoid factor activity at a 1:81920 titer. BJκ-type protein and Aκ paraprotein reappeared in her urine. Her femoral lymph node was biopsied, and the histology results were similar to those of the primary biopsy. A histological and cytological examination of bone marrow was unremarkable. She received one course of lomustine, VePesid (etoposide), vinblastine, and dexamethasone (CEVD) chemotherapy, which produced an effect. However, she refused any further treatment.

After 19 months, she again complained of progressive weakness and weight loss. On examination, generalized enlargement of peripheral and visceral (up to 6.0 cm diameter) lymph nodes, her liver, and her spleen were found. Monoclonal IgAκ secretion reached a level of 44.5 g/L, and BJκ-type protein was found in her urine. A myelogram revealed 1.0% plasma cells and 18% lymphocytes. After one course of doxorubicin, cyclophosphamide, vincristine, VePesid (etoposide), and prednisolone (CHOEP) chemotherapy, she refused further treatment. No chemotherapy was provided subsequently.

Over the next 16 years, a gradual reduction of her peripheral and visceral lymph nodes to normal size was noted. The lymph node size reduction correlated with a decrease in paraprotein levels to the current undetectable value (the changes in paraprotein levels are shown in Table 1). A normal level of immunoglobulins was restored.

**Table 1:** Paraprotein levels during the observation period

	10/1998	05/1999 (after 9 × CHOP)	09/1999 progression	07/2000 progression	2004	2007	2009	2011	2015
PIgAκ (g/l)	72	traces	37.5	44.5	9.2	traces	traces	–	–
BJκ	+	–	+	+	–	–	–	–	–

*BJκ* Bence-Jones κ-type protein, *CHOP* doxorubicin, cyclophosphamide, vincristine, and prednisolone, *PIgAκ* Aκ paraprotein

The unusual clinical course of the disease has prompted us to exclude the reactive nature of plasma cell infiltration.

We performed polymerase chain reaction (PCR) of slices from paraffin blocks to analyze the gene rearrangements of heavy chains and kappa light chains of immunoglobulins. However, we failed to show monoclonal rearrangements of these genes. At the Department of Pathology, Hematopathology Section and Lymph Node Registry, Christian-Albrechts University Kiel and University Hospital Schleswig-Holstein (Germany), immunohistochemistry of the lymph nodes was repeated, which confirmed the monotypic positivity of the plasma cells for the kappa light chains of immunoglobulins.

## Discussion

PLNP can be diagnosed only after exclusion of the terminal progression of multiple myeloma or metastatic primary EMP. The lack of bone destruction and plasma cell infiltration of bone marrow enabled us to confidently exclude multiple myeloma. Additional supporting evidence was found in the absence of CD56 plasma cells expression.

EMP of the upper respiratory tract constitutes 76 to 82% of all cases of EMP, and it metastasizes to the cervical lymph nodes in approximately 15% of cases [1]. However, in this case, the lymph node lesion was generalized and exhibited no evidence of upper respiratory tract plasmacytoma.

The differential diagnosis of PLNP and nodal marginal zone lymphoma (MZL) with extensive plasmacytic differentiation is difficult. In 1999, Hussong *et al*. published an article that suggested that EMP is in most cases MZL with severe plasmacytic differentiation and, in particular, that PLNP is a nodal MZL with pronounced plasma cell differentiation [23]. Later, cases of mucosa-associated lymphoid tissue (MALT) lymphoma (extranodal MZL) of different locations that transformed into plasmacytoma were described [24, 25].

Among autoimmune diseases, SS has the highest incidence of malignant lymphoproliferative transformation. Therefore, SS has been considered a crossroad between the autoimmune and lymphoproliferative disorders [26]. Voulgarelis *et al*. analyzed 54 cases of lymphoma in patients with SS and found MZL in 74% [27]. In our cohort of patients with SS and lymphoma, MZL was also present in 83% (106/128 patients). However, development of PLNP in patients with SS is extremely rare [4, 20]. The fact that our patient had SS prompted us to critically evaluate the morphological characteristics of her lymph nodes to eliminate nodal MZL with extensive plasmacytic differentiation.

Despite examination of her lymph nodes from three different anatomical regions, we could not detect any morphological signs of nodal MZL. Her lymph nodes contained only small remnants of B cell follicles without germinal centers. While the interfollicular areas were expanded and consisted of a monomorphic infiltrate of mature plasma cells (Marschalko type), centrocyte-like and monocytoid cells were absent.

Du *et al*. described a rare disease, so-called HHV-8-associated and EBV-associated germinotropic lymphoproliferative disorder, in which the monotypic light chain immunoglobulins can be found in plasmablasts. However, an analysis of the immunoglobulin gene by PCR showed an oligoclonal or polyclonal pattern [28]. Despite the monotypic expression of IgAκ by plasma cells, we also were unable to confirm the presence of clonality by gene rearrangement of immunoglobulin heavy and light chains. Shao *et al*. showed clonal *IGH* and/or *IGK* gene rearrangements in PCR analysis in only 64% (7/11) of the cases of plasmacytoma, despite the apparent light chain restriction found by immunohistochemistry [4]. It is possible that the inability to demonstrate the clonality of the heavy and light chain gene rearrangements of immunoglobulins in plasmacytomas is due to somatic hypermutations occurring in primer binding sites.

IgA plasmacytomas exhibit several distinctive features. These include a more common presentation in patients less than 30 years of age, a more common presentation in nodal rather than extranodal sites, frequent involvement of multiple lymph node sites rather than solitary lesions, absence of progression to plasma cell myeloma, and evidence of immune system dysfunction [4]. Our case of IgA-secreting plasmacytoma largely confirms the findings of these authors, with multiple lymph node lesions, an absence of progression to multiple myeloma, and plasmacytoma development against a background of the long-term course of SS.

Despite the retention of PLNP in the World Health Organization (WHO) Classification of Lymphoid Tumors 2008 [29], the disease has practically disappeared from the literature in recent years. It is possible that pathologists who examine the morphological characteristics of lymph node plasmacytoma tend to attribute it in most cases to nodal MZL with pronounced plasma cell differentiation. The ability of plasma cell differentiation is a characteristic feature of MZL. However, currently, no cytogenetic, molecular, or immunophenotypic markers are available to conclusively show that PLNP is a variant of MZL.

The course of plasma cell neoplasia in our patient seems highly unusual. Despite the achievement of complete clinical remission after nine courses of CHOP chemotherapy, the trace paraprotein secretion remained, indicating a residual tumor. The rapid growth of the tumor mass with an increase in paraprotein levels 3 months after completion of therapy demonstrated the aggressive nature of the lymphoma. However, thereafter, the lymphoma gradually underwent a complete clinical remission with the disappearance of paraprotein despite the absence of adequate therapy. The SR of tumor has been noted in a variety of neoplastic conditions. In non-Hodgkin’s lymphoma, this phenomenon has been predominantly reported in indolent histologic subtypes. The reasons for SRs have not been identified and there may be different reasons in each case. It is well known that the disappearance of the stimulating antigen can lead to the disappearance of the tumor. Thus, elimination of *Helicobacter pylori* can cause regression of gastric MALT lymphoma. We can assume that our patient had an antigen (virus?), and its spontaneous elimination led to regression of plasmacytoma. Another possible mechanism is recapture of immunoregulatory control. Cases are described of EMP-like post-transplantation lymphoproliferative disorder, including those with lymph nodes involvement, which regressed after reduction of immunosuppression [30, 31]. It is possible that the cessation of chemotherapy in our patient could have led to the restoration of immune control and subsequent tumor regression.

## Conclusions

Despite the current tendency to consider PLNP as a nodal MZL with extensive plasmacytic differentiation, critical histological and immunohistochemical evaluation of three lymph node biopsies from different anatomical areas at different times showed no signs of nodal MZL. An 18-year follow-up of our patient with PLNP demonstrated an extremely unusual clinical course. Initially, PLNP was refractory to chemotherapy, but the plasmacytoma subsequently underwent a complete spontaneous remission.

## Abbreviations

BJ: Bence-Jones
CEVD: Lomustine, VePesid (etoposide), vinblastine, and dexamethasone
CHOEP: Doxorubicin, cyclophosphamide, vincristine, VePesid (etoposide), and prednisolone
CHOP: doxorubicin, cyclophosphamide, vincristine and prednisolone
EBER: Epstein–Barr virus-encoded small RNA
EBV: Epstein–Barr virus
EMP: Extramedullary plasmacytoma
HHV-8: Human herpesvirus type 8
HIV: Human immunodeficiency virus
MALT: Mucosa-associated lymphoid tissue
MZL: Lymphoma of marginal zone cells
PCR: Polymerase chain reaction
PLNP: Primary lymph node plasmacytoma
SR: Spontaneous regression
SS: Sjögren’s syndrome
WHO: World Health Organization
